# Incidence and risk factors of severe acute high-altitude illness in healthy adults first entering the northern Tibetan Plateau of over 5,000 m

**DOI:** 10.3389/fpubh.2024.1400236

**Published:** 2024-09-09

**Authors:** Chun Gao, Guo-Dong Qi, Dan Wang, Zhao-Hui Zhang, Zhong-Xing Liu, Rui-Dong Ge, Zong Yong, Li-E Yan

**Affiliations:** ^1^Department of Gastroenterology, China-Japan Friendship Hospital, Beijing, China; ^2^Research Center for Physical Fitness at High Altitude, Chengguan District Culture and Tourism Bureau, Lhasa Tibet, China; ^3^Department of Quality, Health, Safety and Environmental Protection, China National Petroleum Corporation, Beijing, China; ^4^Evaluation Research Center, Renmin University of China, Beijing, China; ^5^Endoscopy Center, Liaoyang Gastroenterological Hospital, Liaoyang, China; ^6^Department of Rehabilitation Medicine, China-Japan Friendship Hospital, Beijing, China; ^7^Center for Disease Control and Prevention, Shuanghu County Health Commission, Nagqu Tibet, China; ^8^Nursing Department, China-Japan Friendship Hospital, Beijing, China

**Keywords:** acute high-altitude illness, risk factor, northern Tibetan Plateau, healthy adults, incidence

## Abstract

**Background:**

Our study was designed to determine the incidence and risk factors of severe acute high-altitude illness (AHAI) in healthy adults first entering the northern Tibetan Plateau of over 5,000 m.

**Methods:**

In our prospective observational study, we enrolled 500 people who were scheduled for fast ascension to the northern Tibetan Plateau. The primary outcome variable was severe AHAI, defined as the presence of serious symptoms that could not be ameliorated by general treatment and required evacuation to lower altitudes. According to the inclusion and exclusion criteria, a cohort of 383 healthy people was included in the statistical analysis. We calculated the incidence of severe AHAI, identified the risk factors, and the differences in the most severe symptoms experienced.

**Results:**

Sixty-eight people were diagnosed with severe AHAI, and the incidence was 17.8%. Compared to individuals without severe AHAI, those with severe AHAI were more likely to be over the age of 40 years, of Han Chinese nationality, and living at an altitude of <1,500 m. They were less likely to belong to the Yi nationality, had a lower altitude of permanent residence, and exhibited decreased levels of lymphocyte count and hemoglobin concentration. Multivariable logistic regression showed that the mean altitude of permanent residence [per kilometer, adjusted odds ratio (AOR) = 0.464; 95% confidence interval (CI), 0.304–0.708; *p* < 0.001] and lymphocyte count (AOR = 0.606; 95% CI, 0.378–0.970; *p* = 0.037) were the independent risk factors. Headache and dyspnea ranked in the top two of the most severe symptoms for people with severe AHAI.

**Conclusion:**

Living at lower altitudes and having a decreased lymphocyte level were the risk factors of severe AHAI in healthy adults first entering the plateau of over 5,000 m.

## 1 Introduction

The northern Tibetan Plateau, which is also named the “Qiangtang Plateau,” is the highest altitude region of the Qinghai-Tibetan Plateau, covering an area of approximately 0.6 million square kilometers with an average elevation of over 4,500 m and an annual mean temperature below 0°C (1–3). The climate characteristics of this plateau were severe hypoxia, extreme cold, strong ultraviolet radiation exposure, and extremely low absolute humidity (4–6). Permanently living in or temporarily traveling to high altitudes may induce a series of physiological and pathological changes in humans, and the main affecting factor is hypobaric hypoxia (4, 7). When people enter an altitude of over 3,000 m, they usually begin to develop symptoms of hypoxia, for example, headache, dyspnea, insomnia, fatigue, abdominal distension, and loss of appetite (8, 9).

AHAI comprises acute mountain sickness (AMS), high-altitude cerebral edema, and pulmonary edema. AMS is the most common type of AHAI, affecting more than 25% of individuals after exposure to altitudes above 3,500 m and over 50% of individuals at altitudes of 6,000 m (10, 11). Although AMS is usually self-limited for most people and the symptoms can be relieved by rest and oxygen inhalation, approximately 1% of them can progress to severe ailments such as pulmonary edema and cerebral edema (12, 13). Extensive research has been conducted on the pathophysiological processes, but the precise pathogenesis of AHAI has not been explained clearly (14–17). Hypobaric hypoxia has been taken as the main contributing factor, leading to hyperventilation, fluid retention, inflammation, increased pulmonary artery pressure, and raised intracranial pressure (16, 17).

Recently, there has been a growing trend of people ascending to high altitudes worldwide for tourism, work, sports, or military activities (11, 18). Therefore, to reduce the incidence rate of AHAI, early recognition of risk factors and identification of susceptible individuals at low altitudes are especially important. Some risk factors of AHAI have been identified, for example, the altitude reached, fast ascent, older age, obesity, a history of AMS, and lack of pre-acclimatization (13, 18, 19). Several pieces of literature have found an association between lymphocyte level and high altitude (20–23). One *in vitro* study used isolated peripheral blood lymphocytes as biosensors to test the effect of hypobaric hypoxia and suggested that peripheral blood lymphocytes could be used for monitoring adaptive responses to environmental challenges (20). Another study showed that high altitude (HA) hypobaric hypoxia exacerbates the severity of ulcerative colitis in a mouse model by upregulating CD4+ Th1 and Th 17 lymphocytes (23). In this study, a cohort of people was scheduled for a rapid ascent to the northern Tibetan Plateau, reaching altitudes of over 5,000 m. Our study was designed to determine the incidence and risk factors of severe AHAI in these healthy adults first entering this plateau.

## 2 Materials and methods

### 2.1 Study design and screening flow

**Figure 1:**
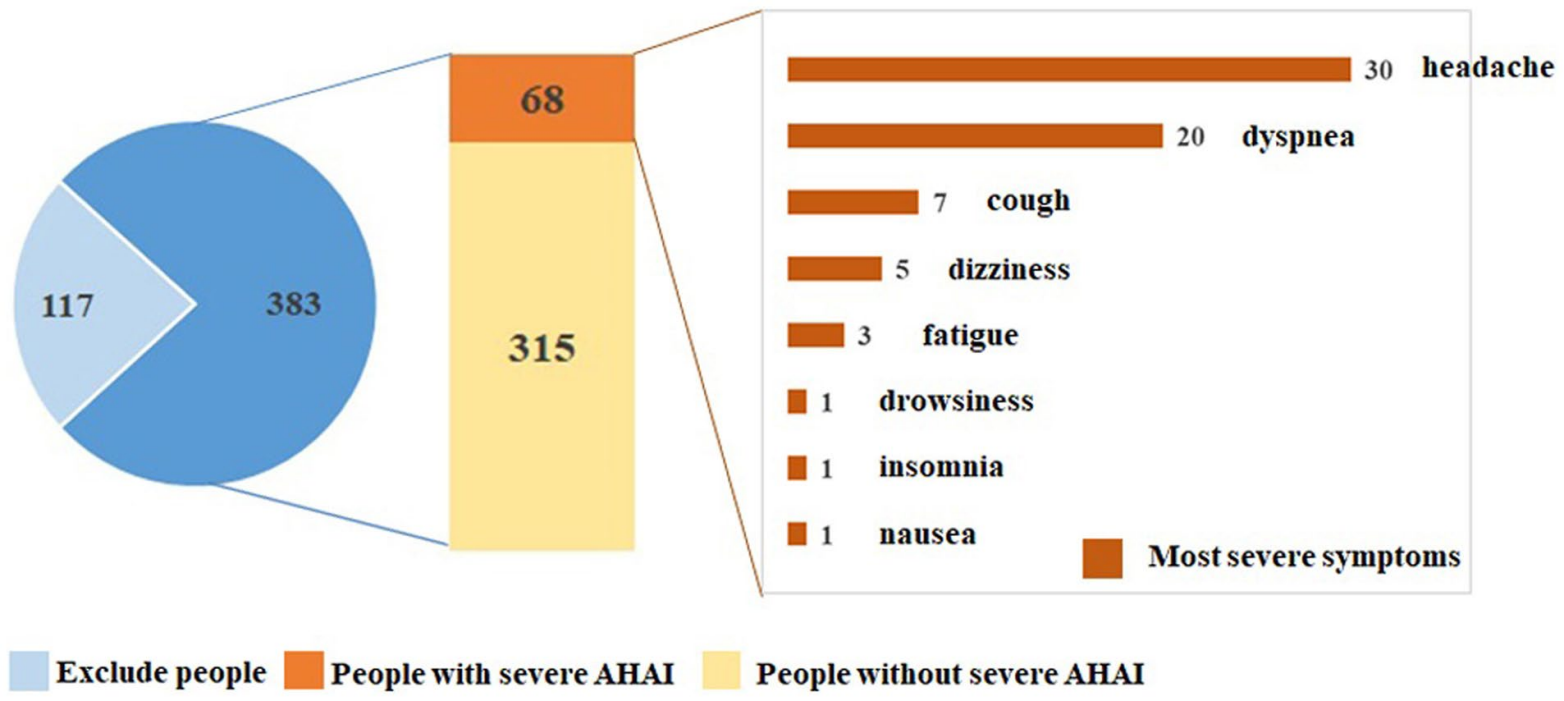
Screening chart of people enrolled in our study. AHAI, acute high-altitude illness.

Shuanghu County of the Tibet Autonomous Region is situated at the northern Tibetan Plateau between north latitude 30–36° and east longitude 83–90°, with an average elevation of over 5,000 m. A cohort of 500 people was scheduled for the assignment to Shuanghu County in 3–5 days. The screening flow is shown in Figure 1. They would be divided into two groups based on the presence or absence of severe AHAI. Our prospective observational study was designed to determine the incidence and risk factors of severe AHAI in healthy adults first entering the plateau of over 5,000 m. Our study was conducted in accordance with the *Declaration of Helsinki* and approved by the Chengguan District Culture and Tourism Bureau, Lhasa, Tibet. Written informed consent was obtained from all the participants.

### 2.2 Study population and physical examination

Five hundred people were enrolled, and finally, a cohort of 383 healthy people was included in the statistical analysis (Figure 1). The inclusion criteria included: (1) age between 18 and 59 years old; (2) never having visited this plateau or entered high-altitude areas of over 5,000 m; (3) not having stayed at high altitudes of over 3,000 m in the past 2 years; and (4) having obtained written informed consent, sufficient data for statistical analysis, and completing follow-up. The exclusion criteria included: (1) physical examination findings that were independently assessed by two team doctors and considered to be unsuitable for entering the plateau of over 5,000 m and (2) a history of serious medical conditions based on their self-reported records. Their physical examinations included demographic data, blood parameters, biochemical parameters, electrocardiogram (ECG), chest computed tomography (CT) scan, echocardiography, lung function test, and skull magnetic resonance imaging (MRI) examination. The examinations were conducted at hospitals near the participants’ permanent residences, and the resulting data were treated as baseline characteristics. The normal reference values of routine blood and liver function tests are listed in Supplementary Table S1.

### 2.3 Definition of severe AHAI and study parameters

Instead of the Lake Louise AMS score (LLS), we gave close attention to the primary outcome. Severe AHAI is defined as people having serious symptoms that cannot be ameliorated by general treatment and required evacuation to lower altitudes. Study parameters are outlined in Tables 1, 2. For age, the mean ± SD and percentage of age over 40 were described. Given that 360 (94.0%) participants were of Han Chinese or Yi nationality, ethnicities were divided into three groups: Yi, Han, and other. The altitudes of permanent residence were split into four groups: <1,500 m, 1,500–1,999 m, 2,000–2,499 m, and 2,500–2,999 m. Routine blood and liver function tests included 10 indices: white blood cell count, lymphocyte count, neutrophil count, hemoglobin, red blood cell count, platelet count, ALT, AST, total bilirubin, and serum albumin.

**Table 1:** Baseline characteristics of our study population.

Characteristics	Study population* (*n* = 383)	People with severe AHAI (*n* = 68)	People without severe AHAI (*n* = 315)	*p*-Value
Mean age, mean ± SD, years	33.8 ± 7.3	34.9 ± 6.8	33.5 ± 7.4	0.161
Age > 40 years, no. (%)	81 (21.1)	21 (30.9)	60 (19.0)	0.030
Age ≤ 40 years, no. (%)	302 (78.9)	47 (69.1)	255 (81.0)	—
Gender, no. (%)				
Male	278 (72.6)	43 (63.2)	235 (74.6)	0.057
Female	105 (27.4)	25 (36.8)	80 (25.4)	—
Ethnicity, no. (%)				
The Yi nationality	239 (62.4)	35 (51.5)	204 (64.8)	0.040
The Han nationality	121 (31.6)	29 (42.6)	92 (29.2)	0.031
Other ethnic groups	23 (6.0)	4 (5.9)	19 (6.0)	1.000
Altitude of permanent residence				
Average, mean ± SD, m	1,802 ± 582	1,569 ± 648	1,852 ± 556	0.001
<1,500 m, no. (%)	59 (15.4)	20 (29.4)	39 (12.4)	<0.001
1,500–1,999 m, no. (%)	219 (57.2)	33 (48.5)	186 (59.0)	0.112
2,000–2,499 m, no. (%)	62 (16.2)	12 (17.6)	50 (15.9)	0.719
2,500–2,999 m, no. (%)	43 (11.2)	3 (4.4)	40 (12.7)	0.050
Body mass index (BMI)				
Average, mean ± SD, kg/m^2^	22.99 ± 3.48	23.27 ± 3.61	22.93 ± 3.45	0.460
Obesity, no. (%)	94 (24.5)	18 (26.5)	76 (24.1)	0.684
Height, mean ± SD, cm	165.9 ± 8.6	164.6 ± 9.2	166.2 ± 8.5	0.161
Weight, mean ± SD, kg	63.5 ± 11.8	63.2 ± 12.4	63.5 ± 11.7	0.867
Blood pressure				
Hypertension, no. (%)	51 (13.3)	13 (19.1)	38 (12.1)	0.120
SBP, mean ± SD, mmHg	122.1 ± 13.2	122.0 ± 13.7	122.1 ± 13.1	0.959
DBP, mean ± SD, mmHg	78.4 ± 9.7	79.2 ± 11.2	78.2 ± 9.4	0.423
Basal heart rate, mean ± SD, bmp	76.6 ± 11.5	77.6 ± 11.0	76.4 ± 11.7	0.418

*A total of 383 people entered the statistical analysis, including 68 (17.8%) people with severe acute high-altitude illness (AHAI) and 315 people without severe AHAI. Severe AHAI is defined as people having serious symptoms that cannot be ameliorated by general treatment, and they must be evacuated to lower altitudes.

AHAI, acute high-altitude illness; bmp, beats per minute; DBP, diastolic blood pressure; SBP, systolic blood pressure.

**Table 2:** Auxiliary examination results of our population.

Auxiliary examinations	Study population (*n* = 383)	People with severe AHAI (*n* = 68)	People without severe AHAI (*n* = 315)	*p*-Value
Routine blood test, mean ± SD				
White blood cell count, ×^9^/L	7.09 ± 1.96	6.97 ± 1.84	7.11 ± 1.98	0.586
Neutrophil count, ×^9^/L	4.21 ± 1.70	4.22 ± 1.48	4.20 ± 1.75	0.939
Lymphocyte count, ×^9^/L	2.28 ± 0.61	2.14 ± 0.53	2.31 ± 0.62	0.039
Red blood cell count, ×^12^/L	5.09 ± 0.55	4.98 ± 0.56	5.11 ± 0.54	0.074
Hemoglobin, g/L	158.6 ± 18.4	154.2 ± 21.7	159.6 ± 17.5	0.027
Platelet count, ×^9^/L	237.0 ± 61.7	238.2 ± 67.3	236.7 ± 60.6	0.855
Liver function test, mean ± SD				
ALT, Median (Q1–Q3), U/L	23 (16–38)	22 (16–44)	23 (17–37)	0.872
AST, Median (Q1–Q3), U/L	22 (18–30)	21 (18–31)	23 (19–30)	0.879
Total bilirubin, μmol/L	13.89 ± 6.62	14.25 ± 7.85	13.81 ± 6.36	0.654
Serum albumin, g/L	47.15 ± 3.80	46.78 ± 3.61	47.24 ± 3.84	0.537
Electrocardiographic examination				
Normal, no. (%)	260 (67.9)	40 (58.8)	220 (69.8)	0.078
Marginal ECG, no. (%)	123 (32.1)	28 (41.2)	95 (30.2)	—
Normal for examinations, no. (%)				
Echocardiography	383 (100.0)	68 (100.0)	315 (100.0)	—
Chest CT scan	383 (100.0)	68 (100.0)	315 (100.0)	—
Lung function test	383 (100.0)	68 (100.0)	315 (100.0)	—
Skull MRI examination	383 (100.0)	68 (100.0)	315 (100.0)	—

AHAI, acute high-altitude illness; ALT, alanine aminotransferase; AST, aspartate aminotransferase; CT, computed tomography; ECG, electrocardiogram; MRI, magnetic resonance imaging.

### 2.4 Treatment of severe AHAI and follow-up

Our study participants were accompanied by two team doctors who could help to treat the possible AHAI. After the people entered the northern Tibetan Plateau, they were asked to monitor their heart rates, blood pressure, fingertip arterial oxygen saturation (SaO_2_), and the top three cardinal symptoms. During the first week, monitoring was conducted three times a day, with results reported to the team doctors. If participants exhibited serious symptoms, the doctors could provide appropriate treatments, such as oxygen inhalation, sleep medications, and painkilling drugs. Those did not experience sufficient relief after these treatments were rushed to Shuanghu County Hospital. After diagnosis and general treatment, individuals with severe AHAI were evacuated to lower altitudes and categorized as the case group.

The follow-up lasted for at least 4 weeks. Individuals with severe AHAI were followed up by investigators at least once a week and for a minimum of 1 month via phone. During the follow-up period, cardinal symptoms, the progression of AHAI, recovery, and prognosis were recorded. For individuals without severe AHAI, the contents of monitoring remained unchanged, and the frequency of monitoring decreased gradually. The frequency was twice a day in the second week and once a day in the next 2 weeks.

### 2.5 Statistical analysis

SPSS 23.0 for Windows (Chicago, IL, USA) was used for the statistical analysis. Based on the variable types, data were described as the mean ± standard deviation, median (interquartile range), or number and proportion. To compare the baseline characteristics and results of auxiliary examinations, Student’s t-test, the Mann–Whitney U-test, and the chi-square test were used for continuous variables with normal distribution, continuous variables with skewed distribution, and categorical variables, respectively. Univariate and multivariable analyses were conducted using unconditional logistic regression to determine risk factors of severe AHAI. Stepwise analysis (backward: Wald; entry: 0.05; removal: 0.10) was adopted. The results were expressed as odds ratios and 95% confidence intervals. The chi-square test was used to compare the differences in the most severe symptoms between the two groups.

## 3 Results

### 3.1 People’s baseline characteristics and auxiliary examination results

Figure 1 shows the screening flow and 500 people were enrolled. Based on the inclusion and exclusion criteria, a cohort of 383 healthy people was included in the statistical analysis. Their baseline characteristics and auxiliary examination results are listed in Tables 1, 2. The mean age was 33.8 ± 7.3 years and 81 (21.1%) participants were over 40 years. Two hundred and seventy-eight (72.6%) participants were male and 360 (94.0%) participants were Han Chinese or Yi nationality. For the altitude of permanent residence, the mean altitude was 1,802 ± 582 m, with 219 participants living at altitudes of 1,500–1,999 m. Routine blood tests revealed average lymphocyte counts of 2.28 ± 0.61 × ^9^/L and hemoglobin levels of 158.6 ± 18.4 g/L. All participants had normal results from echocardiography, chest CT scan, lung function, and skull MRI examinations.

### 3.2 Incidence of participants diagnosed with severe AHAI

Sixty-eight participants diagnosed with severe AHAI were evacuated to the lower altitudes, and the incidence of severe AHAI was 17.8% (Figure 1). Among these 68 participants, the mean age was 34.9 ± 6.8 years, and 25 (36.8%) participants were female. For ethnicity, 35 (51.5%) participants were of the Yi nationality and 29 (42.6%) were of the Han Chinese nationality. Twenty (29.4%) participants lived at altitudes of less than 1,500 m. The results of routine blood and liver function tests are shown in Table 2. Electrocardiographic examination showed that 28 (41.2%) participants were diagnosed with marginal ECG.

### 3.3 Comparative analysis between participants with and without severe AHAI

Using Student’s t-test, the chi-square test, and the Mann–Whitney U-test, a comparative analysis of baseline characteristics and auxiliary examination results between participants with and without severe AHAI are shown in Tables 1, 2. Compared to those without severe AHAI, those with severe AHAI had higher percentages of age over 40 (30.9% vs. 19.0%, *p* = 0.030), Han Chinese nationality (42.6% vs. 29.2%, *p* = 0.031), and living at an altitude of <1,500 m (29.4% vs. 12.4%, *p* < 0.001), a lower percentage of Yi nationality (51.5% vs. 64.8%, *p* = 0.040), a lower altitude of permanent residence (1,569 ± 648 m vs. 1,852 ± 556 m, *p* = 0.001), and decreased levels of lymphocyte count (2.14 ± 0.53 × ^9^/L vs. 2.31 ± 0.62 × ^9^/L, *p* = 0.039) and hemoglobin concentration (154.2 ± 21.7 g/L vs. 159.6 ± 17.5 g/L, *p* = 0.027).

### 3.4 Risk factors of severe AHAI by univariate analysis

Logistic regression analysis, including both univariate and multivariable analyses, was used to determine risk factors. Based on the results of the comparative analysis (Tables 1, 2), 10 variables were considered potential predicting factors. The univariate analysis (Table 3) showed that seven variables had significant differences, including age > 40 years (OR = 1.899; 95% CI, 1.057–3.413; *p* = 0.032), the Yi nationality (OR = 0.577; 95% CI, 0.340–0.979; *p* = 0.042), the Han nationality (OR = 1.802; 95% CI, 1.052–3.088; *p* = 0.032), the mean altitude of permanent residence (OR = 0.469; 95% CI, 0.308–0.713; *p* < 0.001), living at the altitude of <1,500 m (OR = 2.949; 95% CI, 1.586–5.482; *p* = 0.001), lymphocyte count (OR = 0.617; 95% CI, 0.389–0.979; *p* = 0.040), and hemoglobin concentration (OR = 0.985; 95% CI, 0.971–0.998; *p* = 0.028).

**Table 3:** Risk factors of severe AHAI by univariate analysis.

Variables	OR	95% CI	*p*-Value
Age > 40 years	1.899	1.057–3.413	0.032
Gender: Male	0.586	0.336–1.019	0.058
Ethnicity: the Yi nationality	0.577	0.340–0.979	0.042
Ethnicity: the Han nationality	1.802	1.052–3.088	0.032
Mean altitude of permanent residence (Km)	0.469	0.308–0.713	<0.001
Altitude of permanent residence: <1,500 m	2.949	1.586–5.482	0.001
Lymphocyte count	0.617	0.389–0.979	0.040
Red blood cell count	0.644	0.397–1.046	0.075
Hemoglobin	0.985	0.971–0.998	0.028
Marginal electrocardiogram	1.621	0.945–2.781	0.079

AHAI, acute high-altitude illness; CI, confidence interval; OR, odds ratio.

### 3.5 Multivariable analysis for risk factors of severe AHAI

**Figure 2:**
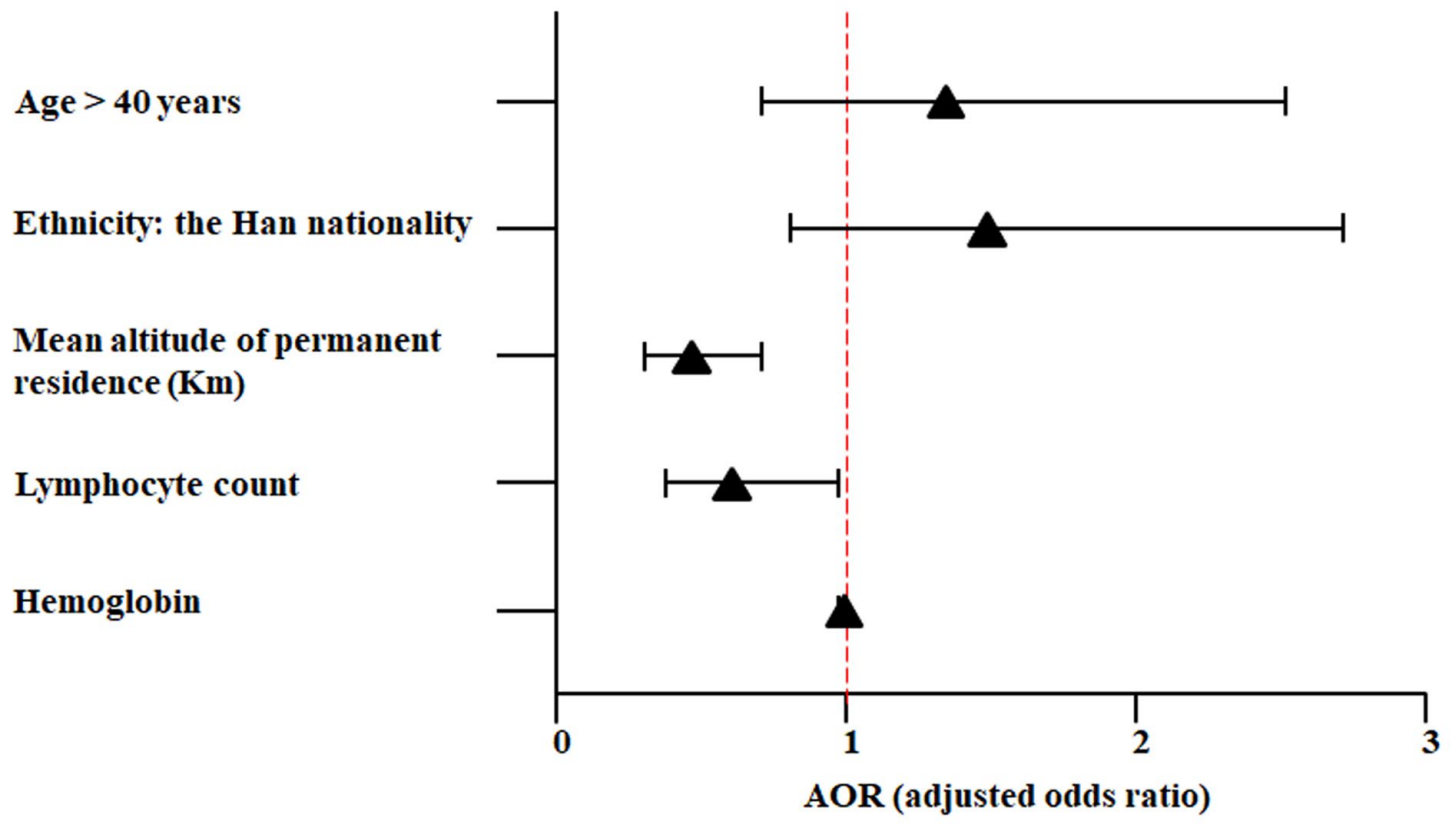
Risk factors of severe AHAI by multivariable analysis.

Since the Yi nationality and living at an altitude of <1,500 m were repetitive variables, they were excluded from the final analysis. Five variables were included in the multivariable analysis: age > 40 years, Han nationality, mean altitude of permanent residence (in kilometers), lymphocyte count, and hemoglobin concentration (Figure 2 and Table 4). The results showed that only two variables had statistical differences, including the mean altitude of permanent residence (per kilometer, AOR = 0.464; 95% CI, 0.304–0.708; *p* < 0.001) and lymphocyte count (AOR = 0.606; 95% CI, 0.378–0.970; *p* = 0.037), indicating that living at lower altitudes and having a decreased lymphocyte level were the risk factors of severe AHAI in healthy adults first entering the plateau of over 5,000 m. A comparative analysis of routine blood and liver function tests at different altitude levels is shown in Supplementary Table S2.

**Table 4:** Multivariable analysis for risk factors of severe AHAI.

Variables	AOR	95% CI	*p*-Value
Age > 40 years	1.339	0.712–2.515	0.365
Ethnicity: the Han nationality	1.482	0.810–2.712	0.202
Mean altitude of permanent residence (Km)	0.464	0.304–0.708	<0.001
Lymphocyte count	0.606	0.378–0.970	0.037
Hemoglobin	0.989	0.975–1.003	0.128

AOR, adjusted odds ratio; CI, confidence interval.

### 3.6 Most severe symptoms between participants with and without severe AHAI

**Figure 3:**
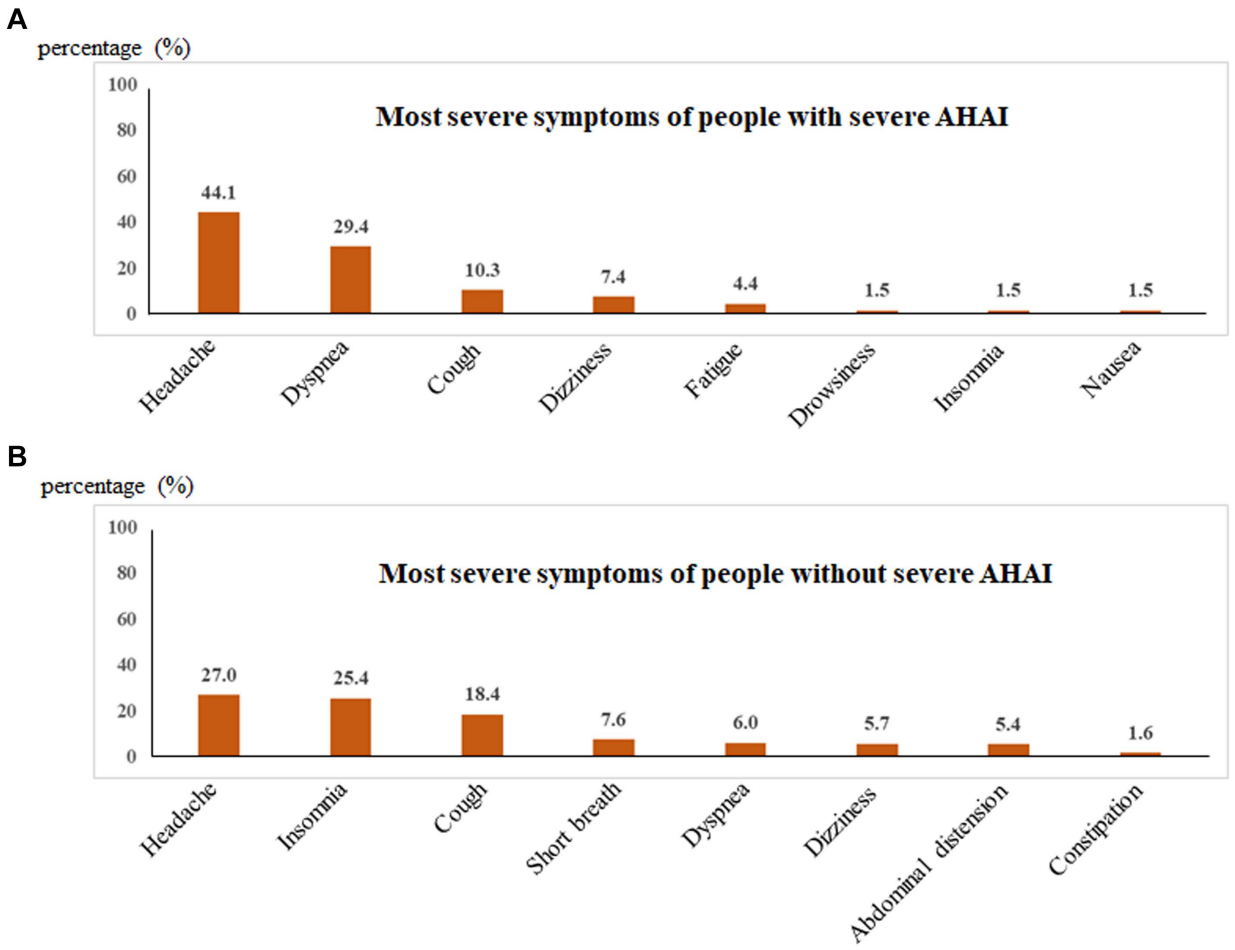
Percentages of most severe symptoms of people with and without severe AHAI.

The most severe symptoms were recorded, including headache, dyspnea, dizziness, cough, fatigue, drowsiness, insomnia, nausea, abdominal distension, constipation, shortness of breath, anorexia, and toothache. For participants with severe AHAI, the top five most severe symptoms were headache (44.1%), dyspnea (29.4%), cough (10.3%), dizziness (7.4%), and fatigue (4.4%), whereas for the participants without severe AHAI, they were headache (27.0%), insomnia (25.4%), cough (18.4%), shortness of breath (7.6%), and dyspnea (6.0%) (Table 5 and Figure 3). Headache and dyspnea ranked in the top two for participants with severe AHAI and the percentages were higher than those without severe AHAI. The chi-square test showed that significant differences were found in headache (*p* = 0.005), dyspnea (*p* < 0.001), and insomnia (*p* < 0.001) (Table 5).

**Table 5:** Comparative analysis of the most severe symptoms between people with and without severe AHAI.

Symptoms	Study population (*n* = 383)	People with severe AHAI (*n* = 68)	People without severe AHAI (*n* = 315)	*p*-Value
Headache	115 (30.0)	30 (44.1)	85 (27.0)	0.005
Dyspnea	39 (10.2)	20 (29.4)	19 (6.0)	<0.001
Cough	65 (17.0)	7 (10.3)	58 (18.4)	0.106
Dizziness	23 (6.0)	5 (7.4)	18 (5.7)	0.815
Fatigue	5 (1.3)	3 (4.4)	2 (0.6)	0.041
Drowsiness	1 (0.3)	1 (1.5)	—	—
Insomnia	81 (21.1)	1 (1.5)	80 (25.4)	<0.001
Nausea	3 (0.8)	1 (1.5)	2 (0.6)	0.445
Short breath	24 (6.3)	—	24 (7.6)	—
Abdominal distension	17 (4.4)	—	17 (5.4)	—
Constipation	5 (1.3)	—	5 (1.6)	—
Anorexia	3 (0.8)	—	3 (1.0)	—
Toothache	2 (0.5)	—	2 (0.6)	—

## 4 Discussion

With the increasing number of individuals ascending to high altitudes for work, tourism, sports, or military activities, people diagnosed with AHAI are gradually increasing (11, 18). If the susceptible individuals who had the likelihood of developing AHAI could be identified prior to ascending to high altitudes, they could receive intensive monitoring, and preventative treatment, even avoid rapid ascents or entering high altitudes. Our study was designed to determine the incidence and risk factors of severe AHAI in healthy adults first entering the northern Tibetan Plateau. We showed that living at lower altitudes and having a decreased lymphocyte count were independent risk factors.

Shuanghu County is situated in the northern Tibetan Plateau at an average altitude of over 5,000 m. Most humans may develop symptoms of hypoxia at this altitude (10, 11) and usually avoid rapid ascents to this elevation. In this study, 500 people were scheduled for the assignment, fast ascending to this plateau in 3–5 days. This assignment gave us the chance to conduct this prospective study, and the major contribution of our study was that we obtained the incidence and risk factors of severe AHAI in healthy adults upon rapid ascent to over 5,000 m. The identified risk factors can be used to construct a prediction model to identify susceptible individuals, allowing for targeted monitoring and preventative treatment before ascending to higher altitudes, for work, tourism, sports, or military activities. The authors wish that future studies could help to advance these goals.

Currently, the diagnosis of AMS can be assessed using rating scales, such as the LLS, the AMS-Cerebral (AMS-C) score, the clinical functional score (CFS), the visual analog scale (VAS) score, the Chinese AMS score (CAS), and the Hackett clinical score (10, 13, 24). The LLS has been widely used in clinical and research practice and is regarded as a gold standard in the diagnosis of AMS (10). One study included 143 young Chinese men and found that the prevalence of AMS was 54% after exposure at 3,800 m (13). Another study showed that the prevalence of AMS was 14.8 and 10.1% at 3,650 m and 21.9 and 15% at 4,559 m, assessed using the LLS and AMS-C scores, respectively (24). The prevalence of AMS varied significantly across different populations and rating scales, primarily due to the fact that these rating scales were established on the grounds of subjective self-evaluation. In our study, we used an objective outcome variable, defining severe AHAI as serious symptoms that cannot be ameliorated by general treatment and require evacuation to lower altitudes. The incidence of severe AHAI was 17.8%.

Some risk factors of AHAI have been identified, for example, older age, fast ascent, obesity, the altitude reached, lack of pre-acclimatization, and history of AMS (13, 18, 19). One study was designed to explore the relationship between anxiety, sleep quality, and AMS and found that the Zung Self-Rating Anxiety Scale (SAS) score and Athens Insomnia Scale (AIS) score were risk factors for AMS (13). However, the risk factors of AHAI may be different in different populations (18, 25). Tang et al. found that older age is an independent risk factor for AMS, whereas Small et al. showed that older age was not associated with AMS. Using univariate analysis, our study showed that older age, Han or Yi nationality, the altitude of permanent residence, lymphocyte count, and hemoglobin concentration were the potential risk factors of severe AHAI.

Having a decreased level of lymphocyte count was the first independent risk factor for severe AHAI identified in our study. Past clinical research has found that lymphocyte count, especially the neutrophil-to-lymphocyte ratio (NLR), is associated with high altitude and AHAI (26–28). He et al. found that the incidence of myocardial injury was increased at high altitudes and NLR was an independent risk factor for myocardial injury (26). Nguyen et al. described that exposure to high altitudes induced increased granulocyte-to-lymphocyte ratio and monocyte-to-lymphocyte ratio (27). Vélez-Páez et al. included 340 patients in Quito-Ecuador (2,850 m) and showed that NLR was an independent predictor of mortality in critically ill obese patients (28).

Several basic studies have revealed the potential underlying mechanisms between lymphocytes and high altitude (20–23). One *in vitro* study used isolated peripheral blood lymphocytes to test the effect of HA hypobaric hypoxia and revealed that 3 weeks of exposure to a high altitude of 5,000 m could increase intracellular Ca(2+) concentration and intracellular levels of reactive oxygen species (ROS) and decrease mitochondrial membrane potential in lymphocytes (20). Another study discovered that T helper 2 (Th2) lymphocytes could promote erythropoiesis under the hypoxic conditions of HA (21). The authors further demonstrated that Th2 lymphocytes from hypoxic mice could promote the erythroid differentiation of hematopoietic stem/progenitor cells and the growth of erythroblasts via elevated secretion of interleukin-9 and activin A (21).

Considering that a decreased level of lymphocyte count is associated with severe AHAI in our study, we tried to use the ROC curve and Youden index to determine the range or level of lymphocytes that are considered indicative of severe AHAI. Unfortunately, no optimal cutoff point was obtained, and we wish that it could be resolved by in-depth research in the future. Based on current research, combined with the mean and median of lymphocytes, the value of 2.20 × ^9^/L is recommended as the temporary threshold, indicating that people with lymphocytes <2.20 × ^9^/L have an increased risk of severe AHAI. Identifying susceptible individuals based on lymphocyte count may still be challenging. More in-depth studies are required to resolve this issue.

Living at lower altitudes was the second independent risk factor of severe AHAI in our study. This result was supported by some previous studies, which suggested that residence at moderate altitude could reduce the risk of suffering AHAI during rapid ascent to higher altitude (29, 30). Some limitations in our study should be acknowledged. The first limitation was that our population was from one single center. The potential impact on generalizability should be considered, and the results should be validated with more people. The second was that the research data were obtained mainly from physical examination data and some new biomarkers were not included. The authors wish that easily available variables can be identified and used widely for risk prediction.

In conclusion, our study showed that living at lower altitudes and having a decreased level of lymphocyte count were the risk factors for severe AHAI in healthy adults first entering this Tibetan Plateau of over 5,000 m. Early identification and management of the risk factors for severe AHAI are especially important in clinical practice and public health interventions. Future research should focus on validating findings in diverse populations and exploring additional biomarkers for predicting AHAI risk.

## Data Availability

The original contributions presented in the study are included in the article/Supplementary material, further inquiries can be directed to the corresponding author.
