# Supplementary material for: Incidence and risk factors of severe acute high-altitude illness in healthy adults first entering the northern Tibetan Plateau of over 5,000 m
Source: Front Public Health. 2024 Sep 9;12:1400236. doi: 10.3389/fpubh.2024.1400236 (PMC11420918; doi:10.3389/fpubh.2024.1400236)
Supplement: Supplementary file 1 [file Table_1.DOC]

**Supplementary Table S1**

**The normal reference values of routine blood and liver function tests**

| **Parameter** | **Normal reference value** |
| --- | --- |
| Routine blood test |  |
| White blood cell count | 3.5-9.5 ×9/L |
| Neutrophil count | 1.8-6.3 ×9/L |
| Lymphocyte count | 1.1-3.2 ×9/L |
| Red blood cell count | 4.3-5.8 ×12/L |
| Hemoglobin | 130-175 g/L |
| Platelet count | 125-350 ×9/L |
| Liver function test |  |
| ALT (alanine aminotransferase) | 0-40 U/L |
| AST (aspartate aminotransferase) | 0-42 U/L |
| Total bilirubin | 3.0-22.0 µmol/L |
| Serum albumin | 35-55 g/L |

**Supplementary Table S2 Comparative analysis of routine blood and liver function tests at different altitude levels**

| **Auxiliary examinations** | **< 1500 m (n=59)** | **1500-1999 m**  **(n=219)** | **2000-2499 m**  **(n=62)** | **2500-2999 m**  **(n=43)** | ***P* Value** |
| --- | --- | --- | --- | --- | --- |
| Routine blood test, mean ± SD |  |  |  |  |  |
| White blood cell count, ×9/L | 6.84 ± 1.56 | 7.18 ± 2.17 | 7.30 ± 1.71 | 6.66 ± 1.59 | 0.234 |
| Neutrophil count, ×9/L | 4.16 ± 1.29 | 4.30 ± 1.92 | 4.15 ± 1.34 | 3.89 ± 1.49 | 0.515 |
| Lymphocyte count, ×9/L | 2.22 ± 0.55 | 2.26 ± 0.61 | 2.46 ± 0.65 | 2.20 ± 0.58 | 0.078 |
| Red blood cell count, ×12/L | 5.16 ± 0.56 | 5.04 ± 0.54 | 5.07 ± 0.53 | 5.24 ± 0.56 | 0.132 |
| Hemoglobin, g/L | 159.3 ± 17.8 | 155.5 ± 18.0 | 158.5 ± 15.6 | 174.0 ± 17.8 | <0.001 |
| Platelet count, ×9/L | 223.7 ± 65.5 | 246.0 ± 62.4 | 243.6 ± 55.3 | 200.1 ± 44.0 | <0.001 |
| Liver function test, mean ± SD |  |  |  |  |  |
| ALT, Median (Q1-Q3), U/L | 28 (18-43) | 22 (16-38) | 21 (16-31) | 26 (18-43) | 0.239 |
| AST, Median (Q1-Q3), U/L | 25 (20-31) | 22 (18-30) | 20 (17-26) | 27 (20-37) | 0.010 |
| Total bilirubin, µmol/L | 14.88 ± 7.34 | 13.31 ± 5.87 | 13.26 ± 6.33 | 16.49 ± 8.95 | 0.037 |
| Serum albumin, g/L | 45.97 ± 3.59 | 47.50 ± 3.60 | 47.43 ± 4.65 | 46.27 ± 2.87 | 0.219 |
